# Does Workers’ Compensation Status Affect Outcomes after Lumbar Spine Surgery? A Systematic Review and Meta-Analysis

**DOI:** 10.3390/ijerph18116165

**Published:** 2021-06-07

**Authors:** Fabrizio Russo, Sergio De Salvatore, Luca Ambrosio, Gianluca Vadalà, Luca Fontana, Rocco Papalia, Jorma Rantanen, Sergio Iavicoli, Vincenzo Denaro

**Affiliations:** 1 Department of Orthopaedic and Trauma Surgery, Campus Bio-Medico University of Rome, 00128 Rome, Italy; s.desalvatore@unicampus.it (S.D.S.); l.ambrosio@unicampus.it (L.A.); g.vadala@unicampus.it (G.V.); r.papalia@unicampus.it (R.P.); denaro@unicampus.it (V.D.); 2 Department of Occupational and Environmental Medicine, Epidemiology and Hygiene, Italian Workers’ Compensation Authority (INAIL), 00078 Rome, Italy; lfontana73@yahoo.it (L.F.); s.iavicoli@inail.it (S.I.); 3 Department of Public Health/Occupational Health, University of Helsinki, 00250 Helsinki, Finland; jorma.h.rantanen@gmail.com

**Keywords:** disability, insurance, low back pain, lumbar decompression, lumbar fusion, musculoskeletal disorders, occupational health, pain, return to work, satisfaction

## Abstract

Low back pain (LBP) is currently the leading cause of disability worldwide and the most common reason for workers’ compensation (WC) claims. Studies have demonstrated that receiving WC is associated with a negative prognosis following treatment for a vast range of health conditions. However, the impact of WC on outcomes after spine surgery is still controversial. The aim of this meta-analysis was to systematically review the literature and analyze the impact of compensation status on outcomes after lumbar spine surgery. A systematic search was performed on Medline, Scopus, CINAHL, EMBASE and CENTRAL databases. The review included studies of patients undergoing lumbar spine surgery in which compensation status was reported. Methodological quality was assessed through ROBINS-I and quality of evidence was estimated using the GRADE rating. A total of 26 studies with a total of 2668 patients were included in the analysis. WC patients had higher post-operative pain and disability, as well as lower satisfaction after surgery when compared to those without WC. Furthermore, WC patients demonstrated to have a delayed return to work. According to our results, compensation status is associated with poor outcomes after lumbar spine surgery. Contextualizing post-operative outcomes in clinical and work-related domains helps understand the multifactorial nature of the phenomenon.

## 1. Introduction

Musculoskeletal disorders (MSDs) are the highest contributor to global disability and represent a substantial portion of occupational injury claims with a steadily rising incidence [1]. Low back pain (LBP) is the single worldwide leading cause of disability, has a strong relationship with years lived with disability (YLDs) and, since it was first measured in 1990 [1], it is the most common reason for workers’ compensation (WC) claims [2]. It causes limitations of daily activity and work capacity, with high rates of work absenteeism and considerable economic and health consequences, therefore representing a major critical issue in the context of occupational medicine and public health [3]. 

Surgical procedures are quite commonly used as a treatment for LBP unresponsive to conservative treatments or associated with worsening neurological deficits [4]. The success of a surgical intervention in orthopaedic medicine is influenced by several key factors, the most important of which are the appropriateness of the surgical indication and surgeon’s experience with the specific procedure. However, in this regard, the patient’s compensation status has also been suggested as a potential factor influencing surgical outcomes. Indeed, additional elements including demographic and socioeconomic variables, such as lower degree of education, higher body mass index, smoking and lower annual wages, have been described to negatively impact outcomes following surgery [5]. 

In countries with modern social safety and welfare systems, an integrated compensation policy is guaranteed for disabled people or workers who experience accidents at work or occupational diseases. Compensation strategies and methodologies are extremely variable among nations, but commonly all of them provide workers with healthcare services, wage-replacement support, and other social benefits [6]. Usually, a government authority or a private sector organization acting on its behalf, carry out the administrative decision-making process which, after verifying the possession of eligibility criteria for claims, certifies the release of the different compensation services. Compensation approaches can be basically divided into two broad categories: cause-based systems typically require a correlation between occupational risk factors or work environment/activities and the resulting adverse health effects, whereas disability-based approaches provide benefits and services regardless of cause [7]. Therefore, WC benefits support the injured/sick workers by providing temporary aid, although in the most serious cases involving a high disability degree the type of compensation can also be permanent, until they can meet their respective clinical goals and return to work (RTW) as soon as possible with the least amount of disability. In this regard, it is important to note that the ability to RTW is one of the most clinically important outcomes in workers, in association with scores for disability, satisfaction and pain. 

Nevertheless, it should be considered that available literature data provided evidence that the nature of compensation services and related methods of administration might adversely impact on health and work outcomes [8]. Indeed, several studies have demonstrated that receiving WC is associated with a negative prognosis following treatment for a vast range of health conditions [9,10,11,12,13,14]. Moreover, interactions of claimants with compensation authorities are often referred to by workers as stressful experiences that might induce poor mental health [8]. On the other hand, several procedural and bureaucratic features (e.g., delays in the claim processing times, strict and rigid procedures, lack of communication between workers and authorities) of the WC administrative process can increase the disability duration, thus delaying the reintegration of people into the workforce [15].

However, the influence of WC on the treatment of LBP is still controversial. Indeed, only a few studies have analyzed the impact of WC on outcomes after spine surgery, highlighting the importance of considering WC as a determining factor when evaluating outcomes of different spinal procedures [5,6,16]. Indeed, the reported strength of this association has widely varied from odds ratios of 1.31 to 7.22 among published studies [8,17,18]. The purpose of this meta-analysis is to systematically review the literature and analyze the impact of compensation status on lumbar spine surgery outcomes.

## 2. Materials and Methods

We focused our research on studies concerning the effect of WC on outcomes after lumbar spine surgery, comparing them to non-workers’ compensation (NWC) patients. The Preferred Reporting Items for Systematic reviews and Meta-Analyses (PRISMA) guidelines were used to improve the reporting of the review.

### 2.1. Eligibility Criteria

The research question was formulated using a PICOS-approach: Patient (P); Intervention (I); Comparison (C); Outcome (O) and Study design (S). The aim of this systematic review was to select those articles which described “if patients undergoing lumbar spine surgery (P) with a known WC status (I) have worse results in terms of LBP, disability, satisfaction and time to RTW (O) compared to the NWC population (C)”. For this purpose, only randomized control trials (RCT) and non-randomized controlled studies (NRCT) such as prospective (PS), retrospective (RS) observational studies (OS), case-series (CS) and case-control (CC) studies were included.

#### Inclusion and Exclusion Criteria

Inclusion criteria were:

- Peer-reviewed studies of every level of evidence according to the Oxford Classification. We included in our research RCT and NRCT.
- Studies including working patients with no limitations of age and type of work.
- Studies that reported outcomes for patients undergoing any type of surgical procedure involving the lumbar spine.
- Studies that included at least one assessment for each type of outcome (LBP, disability, satisfaction after surgery and RTW). The pain outcome had to be evaluated by one or more of the following scales: numerical pain rating scale (NRS) and visual analogue scale (VAS). The disability outcome needed to be evaluated by one or more of the following scales: Oswestry Disability Index (ODI); 36-Item Short Form Health Survey (SF-36); 12-Item Short Form Health Survey (SF-12); Roland Morris Disability Questionnaire (RMDQ); functional status (FS) and Lumbar Back Outcome Scale (LBOS). RTW was evaluated as the number of patients that went back to their previous working activities at the time of the last follow-up. The satisfaction rate after surgery was assessed in patients as follows: “Excellent”, “Good”, “Almost complete relief”, “Good deal of relief”, and “Satisfied” were considered as satisfactory outcomes, whereas “Fair”, “Poor”, “Only a little relief”, “No relief or worse” and “Unsatisfied” were considered unsatisfactory. Moreover, in studies where the satisfaction rate was expressed in a numeric scale, values between 0 and 4 were considered unsatisfactory, whilst values between 5 and 10 were considered satisfactory.
- Only articles written in English and Italian were included.

We excluded case reports, technical notes, letters to editors, instructional courses, in vitro and cadaver studies, as well as studies including cervical or thoracic spine procedures.

### 2.2. Search

The articles included in the study were screened from inception to May 2020 through a systematic search of Medline, Scopus, CINAHL, EMBASE and CENTRAL databases. For the search strategy we decided to use the following string: (workers compensation [MeSH Terms]) AND ((spine) OR (lumbar) OR (spine surgery)). We used the keywords isolated or combined. We searched for more studies among the reference lists of the selected papers and systematic reviews.

### 2.3. Study Selection

**Figure 1:**
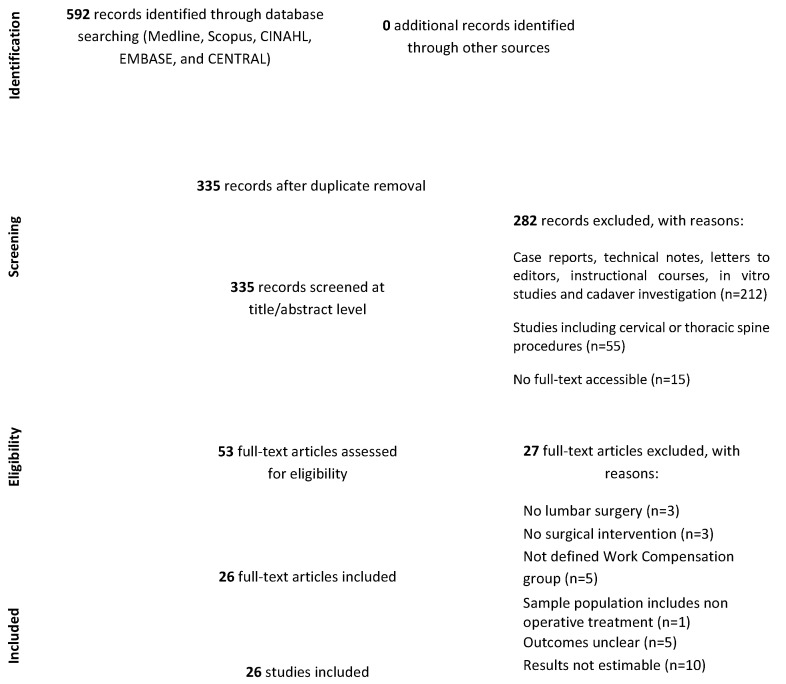
PRISMA flowchart.

We accepted only English and Italian publications. The initial search of the article was conducted by two reviewers (S.D.S. and L.A.). In case of disagreements, the consensus of a third reviewer (F.R.) was asked. The research was conducted using the CADIMA software [19]. The researchers used the following research order: titles were screened first, then abstracts and full papers. A paper was considered potentially relevant, and its full text reviewed, if following a discussion between the two independent reviewers, it could not be unequivocally excluded based on its title and abstract. The full text of all papers not excluded on the basis of abstract or title, was evaluated. The number of articles excluded or included were registered and reported in a PRISMA flowchart (Figure 1). For designing the PRISMA we followed the rules by Moher et al. [20].

### 2.4. Data Extraction

General study characteristics extracted were author, year of publication, country of origin, type of study, level of evidence [21] (LOE), sample size (divided in WC and NWC), mean age (divided in WC, NWC and mean of both groups), last or average follow-up (in case of multiple time points, only the last follow-up was considered), type of surgery, type of comparison group (NWC), outcome measures (LBP, disability, satisfaction and RTW) and differences between groups.

### 2.5. Individual Study Quality

**Figure 2:**
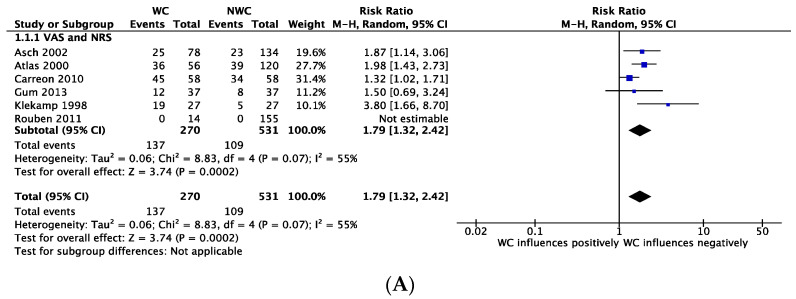

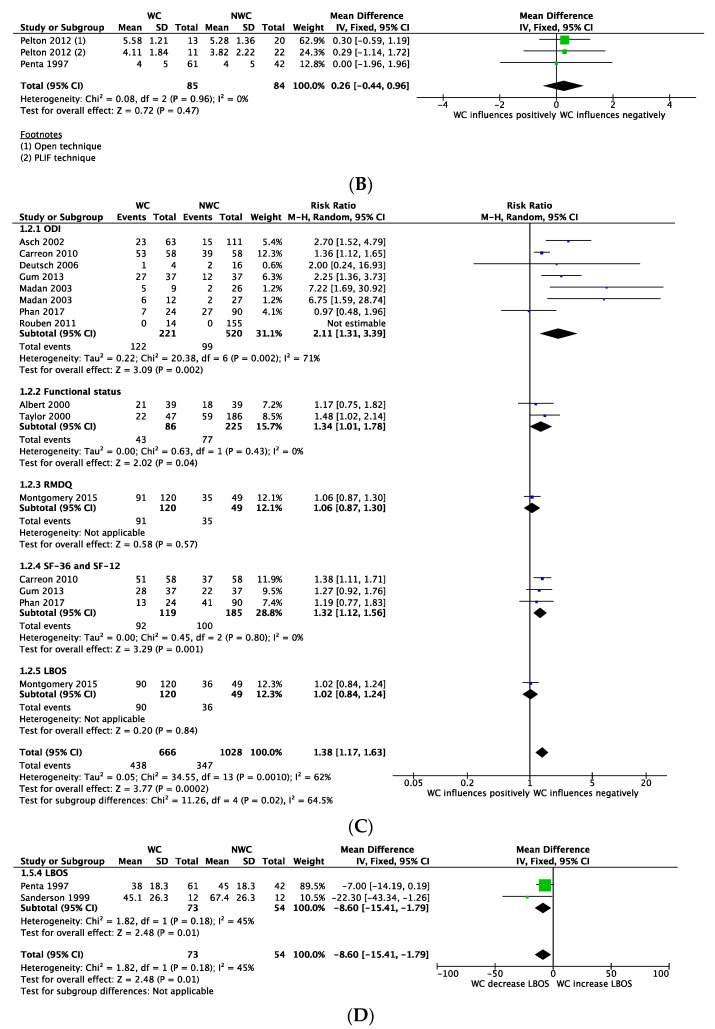
Forest plots depicting the effect of WC on post-operative pain measured by dichotomous (**A**) and continuous data (**B**) and the effect of WC on post-operative disability measured by dichotomous (**C**) and continuous data (**D**).

Given the observational design of included studies, we used the Risk of Bias in Non-randomized Studies of Interventions (ROBINS-I) tool to assess the quality of each study [22] (Figure 2). In order to avoid imprecisions, selected papers were rated independently by two reviewers (S.D.S. and L.A.) and verified by a third one (F.R.).

### 2.6. Quality of Evidence

We used the GRADE approach (Tables S1 and S2) to rate the overall quality of evidence. The GRADE approach classifies the quality of evidence for each outcome by grading the following domains: study design, risk of bias, inconsistency, indirectness, imprecision, publication bias, magnitude of the effect. The quality of evidence was then classified as follows:

- High Quality of Evidence: among 75% of articles included are considered with a low risk bias. Further research is useful to change either the estimate or confidence in results.
- Moderate Quality of Evidence: one of the GRADE domains is not met. Further studies are required to improve the quality of the study and the evidence.
- Low Quality of Evidence: two of the GRADE domains are not met. Further research is critical.
- Very Low Quality of Evidence: three of the GRADE domains are not met. The results of the study are very uncertain. In the case of studies with a sample size inferior to 300 subjects, the quality of the study is considered very low if there was also a high risk of bias (assessed with the ROBINS-I in the present study).

The outcomes assessed were LBP, disability, satisfaction after surgery and RTW evaluated at the end of the treatment. Furthermore, the outcomes were subgrouped per scales. To avoid imprecisions and considering the limited number of studies with continuous data, we considered for GRADE analysis only studies with dichotomous data.

### 2.7. Summary Measures

The summary measures of effect size considered in the study were the risk ratio (RR) for dichotomous data and the mean difference (MD) for continuous variables of data on outcome after surgery in terms of LBP, disability, satisfaction and RTW in WC and NWC populations.

### 2.8. Synthesis of Results

The Mantel–Haenszel method of meta-analysis was performed using Review Manager Software 5.0 (RevMan 5.0, Cochrane Collaborations, London, UK). For dichotomous data, risk ratio was applied using a 5% level of significance. Heterogeneity was assessed by a funnel plot and chi-square test, and inconsistency across studies was quantified using the I^2^ statistic. An I^2^ > 50% or a *p* value of chi-squared test > 0.05 were suggestive of a substantial heterogeneity. Random effects model was used in all analyses.

## 3. Results

### 3.1. Study Selection

We found a total of 592 studies (no additional studies were found in gray literature and no unpublished studies were retrieved). We obtained 335 studies following duplicate removal, 282 of which were excluded through title and abstract screening. Then, 53 full-text articles were screened. Out of these studies, 27 were excluded (no lumbar surgery, *n* = 3; no surgical intervention, *n* = 3; not defined WC group, *n* = 5; sample population including non-operative treatment, *n* = 1; unclear outcomes, *n* = 5 and results not estimable, *n* = 10). After this process, 26 articles were included in our study [16,18,23,24,25,26,27,28,29,30,31,32,33,34,35,36,37,38,39,40,41,42,43,44,45,46].

### 3.2. Study Characteristics

A summary of the characteristics of the included studies is reported in Table 1 and Table 2 (see abbreviations explained below each Table). We did not find any RCTs eligible for this study. The articles selected included 26 NRCT (9 RS, LOE 3; 14 PS, LOE 2; 2 CC, LOE 3 and 1 CS, LOE 4). Studies were published between 1994 [29] and 2017 [40]. A total of 2668 patients (1045 WC and 1623 NWC) were assessed for outcomes after lumbar spine surgery. Of these studies, 3 were performed in Australia, 1 in New Zealand, 1 in Switzerland, 3 in the United Kingdom and 18 in the United States. Pain evaluation in these studies was performed using NRS (3 studies [26,27,32]) and VAS (5 studies [16,25,38,39,41]) scores. The disability outcome was evaluated by one or more of the following scales: ODI (7 studies [16,18,25,27,28,40,41]); SF-36 (2 studies [16,27]); SF-12 (1 study [40]); RMDQ (1 study [36]); FS (2 studies [24,45]) and LBOS (3 studies [36,39,42]). RTW was evaluated in 9 studies [25,26,28,29,33,41,43,44,46] and satisfaction rate in 15 studies [23,25,26,29,30,31,33,34,35,37,39,43,44,45,46]. The studies cited in this review show a moderate heterogeneity between groups (50% < I^2^ < 70%, except for the ODI subgroup with a I^2^ = 71%) and differences in terms of study design, interventions, and outcome variables. Follow-ups were different and ranged from 6 months [38] to 16 years [36].

### 3.3. Methodological Quality

The ROBINS-I tool for NRCT was used to assess the methodological quality of each study. We found 7 studies with an overall risk of bias identified as “low” [18,25,29,30,34,35,40], 14 studies with a “moderate” risk [16,24,26,27,28,31,32,33,38,41,42,43,45,46] and 5 studies with a “serious” risk [23,36,37,39,44]. The quality of evidence of the studies included in the GRADE was classified as “low”. Methodological quality assessments of each study are summarized in Supplementary Figure S1. The quality of evidence of full data was performed using the GRADE approach (Supplementary Tables S1 and S2). The analysis of the data of the study was reported using the RR for studies included dichotomous data and using the MD for studies with continues data. RevMan5 (version 5.3) was used to calculate the RR the MD of the included studies and the heterogeneity between studies using I^2^ and Chi-squared test. The results of the meta-analysis are summarized using forest plots.

### 3.4. Results of Individual Studies

The intervention methods were usually well described in all the included studies. Moderate heterogeneity in the length of follow-up and the surgical procedure were reported in all the studies. We included all types of lumbar spine surgery: discectomy [25,26,31,32,33,34,35,45], laminectomy [31,45], hemilaminotomy [34], lumbar spine fusion [36], minimally invasive surgery or open approach for transforaminal lumbar interbody fusion (TLIF) [16,28,38,41], posterior lumbar interbody fusion (PLIF) [18,23], posterior lumbar fusion (PLF) [18,23,27,30,37,43], anterior lumbar interbody fusion (ALIF) [29,39,40,44], anteroposterior fusion [24] and uninstrumented posterolateral fusion [46]. The authors divided the description of intervention per outcome (LBP, disability, satisfaction rate and RTW). Disability outcomes were subgrouped per measure scale: ODI, SF-12 and SF-36, LBOS and FS. The results of each outcome are reported in Table 2.

### 3.5. Outcome: Pain

Eight observational studies were included (4 PS [25,26,38,39], 2 RS [32,41] and 2 CC [16,27]). They examined the influence of WC on pain modifications in patients undergoing lumbar surgery. Three studies used the NRS scale [26,27,32] and five studies used the VAS scale [16,25,38,39,41] to assess pain. Single studies were assessed for risk of bias using ROBINS-I tool. One study was classified as “serious risk” [39], six as “moderate” [16,26,27,32,38,41] and one as “low risk” [25]. 

The overall quality of evidence in these studies was assessed as “low” according to GRADE. The quantitative effect estimate was reported as RR in studies with dichotomous data (Figure 2A) and as the MD between and within studies (when possible) in case of continuous data (Figure 2B). The overall RR was 1.79, 95% CI 1.32 to 2.42; I^2^ = 55%. 2 studies [38,39] reported the pain outcome as continuous data with a MD between WC and NWC of 0.26, 95% (CI −0.44 to 0.96; I^2^ = 0%), showing a moderate negative influence of WC on pain improvement. 

### 3.6. Outcome: Disability

Twelve observational studies were included (7 PS [18,25,28,36,39,40,45]; three RS [24,41,42] and two CC [16,27]). They examined the influence of WC on disability modifications in patients undergoing lumbar surgery. Seven studies [16,18,25,27,28,40,41] used the ODI scale, two [16,27] used the FS, one [36] used the RMDQ, one [40] used the SF-12, two [16,27] used the SF-36 and three [36,39,42] used the LBOS to assess disability. Single studies were assessed for risk of bias using ROBINS-I tool. Two studies were classified as “serious risk” [36,39], seven as “moderate” [16,24,27,28,41,42,45] and three as “low risk” [18,25,40].

The overall quality of evidence in these studies was assessed as “low” according to GRADE. The quantitative effect estimate was reported as RR in studies with dichotomous data (Figure 2C) and as MD between and within studies (when possible) in case of continuous data (Figure 2D). 

The overall RR was 1.38 (95% CI 1.17 to 1.63; I^2^ = 62%), suggesting an overall negative influence of WC on disability improvement. The ODI subgroup had a RR of 2.11 (95% CI 1.31 to 3.39; I^2^ = 71%); the FS subgroup reported a RR of 1.34 (95% CI 1.01 to 1.78; I^2^ = 0%); the RMDQ subgroup reported a RR of 1.06 (95% CI 0.87 to 1.30; I^2^ = 0%); the SF-12 and SF-36 subgroup showed a RR of 1.32 (95% CI 1.12 to 1.56; I^2^ = 0%); the LBOS subgroup reported a RR of 1.02 (95% CI 0.84 to 1.24; I^2^ = 0%). 2 studies [39,42] reported the disability outcome as continuous data with a MD between WC and NWC of −8.60 (95% CI −15.41 to −1.79; I^2^ = 45%); showing that WC decreased LBOS postoperative values (the lower the value of LBOS, the higher disability of the patient).

### 3.7. Outcome: Return to Work

**Figure 3:**
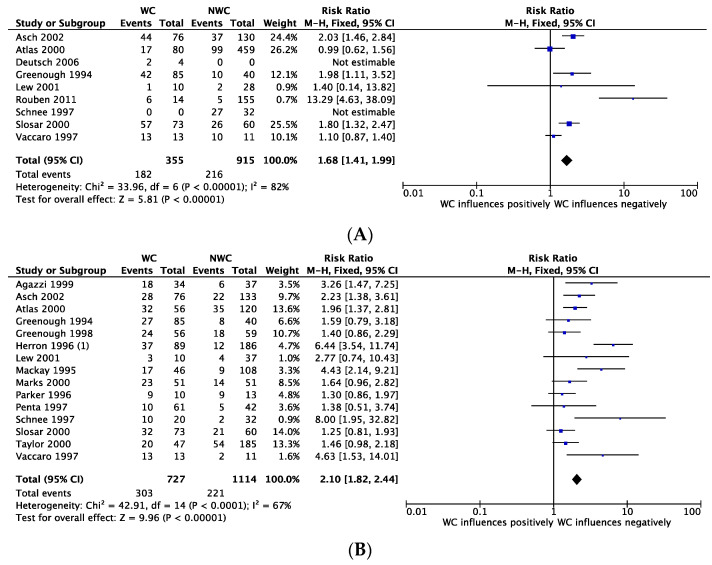
Forest plots depicting the effect of WC on return to work (**A**) and satisfaction (**B**) following lumbar spine surgery.

Nine observational studies were included (four PS [25,26,28,29]; four RS [33,41,43,44] and one CS [46]). They examined how WC influence RTW in patients after lumbar surgery (Figure 3A). RTW was considered at the time of the last follow-up. Single studies were assessed for risk of bias using ROBINS-I tool. One study was classified as “serious risk” [44], 6 as “moderate” [26,28,33,41,43,46] and 2 as “low risk” [25,29]. 

The overall quality of evidence in these studies was assessed as “low” according to GRADE. The quantitative effect estimate was reported as RR. The overall RR was 1.68 (95% CI 1.41 to 1.99; I^2^ = 82%). The studies reported an overall negative influence of WC on RTW in patients after lumbar surgery. 

### 3.8. Outcome: Satisfaction

Fifteen observational studies were included (nine PS [25,26,29,30,31,34,37,39,45]; five RS [23,33,35,43,44] and one CS [46]). They examined the influence of WC on satisfaction modifications in patients undergoing lumbar surgery (Figure 3B). Single studies were assessed for risk of bias using ROBINS-I tool. Four studies were classified as “serious risk” [23,37,39,44], six as “moderate” [26,31,33,43,45,46], and five as “low risk” [25,29,30,34,35]. 

The overall quality of evidence in these studies was assessed as “low” according to GRADE. The quantitative effect estimate was reported as RR. The overall RR was 2.10 (95% CI 1.82 to 2.44; I^2^ = 67%). The studies reported an overall negative influence of WC on satisfaction of patients after lumbar surgery.

## 4. Discussion

The association between compensation status and poor clinical outcomes after orthopaedic surgery has already been described in the literature. In a meta-analysis from Harris et al. [5], WC patients presented with an approximately four times higher odds of worse outcomes after common orthopaedic procedures including shoulder acromioplasty, carpal tunnel release, lumbar fusion and lumbar discectomy compared to NWC patients. Similarly, in a recent meta-analysis from Cheriyan and colleagues [47], outcomes related to patient satisfaction and RTW were investigated in WC and NWC subjects after spine surgery. In this study, authors concluded that WC patients showed a 2.10 RR of unsatisfactory outcomes and a 1.68 RR of delayed RTW after surgical procedures involving the cervical, thoracic, and lumbar spine. These data are congruous with the meta-analysis of de Moraes et al. [48], who reported that compensated patients undergoing lumbar discectomy with or without fusion presented a 1.90 RR of unsatisfactory outcomes after surgery.

In the present study, we analyzed the effect of WC on clinical (pain, disability, and patient satisfaction) and work-related outcomes (RTW) following lumbar spine surgery. Consistently with previous studies, we reported that WC patients tended to exhibit higher post-operative pain (RR = 1.79) and disability (RR = 1.38) as well as lower satisfaction after surgery (RR = 2.10) compared to NWC patients. WC patients demonstrated also a delayed RTW (RR = 1.68) with a significant socioeconomic burden on both work insurances and employers [49]. This latter data is particularly important when considering that the annual expenditure for treating LBP in the United States is greater than $100 billion, with lost wages and reduced productivity accounting for approximately two thirds of the amount [50]. Furthermore, lumbar injuries resulting in spine surgery are among the most expensive WC claims [51]. However, the total cost may not be strictly related to the type of surgery alone but seems also affected by the time between the injury and the surgical treatment. Indeed, Lavin et al. have found that more prolonged and costly WC claims were associated with an interval of more than a year between injury and surgery, hence concluding that timeline of surgical indication is equally important in this subset of patients [52]. 

It is also important to note that several studies have demonstrated that lumbar spine surgery and particularly fusion procedures are characterized by a variable rate of success [53,54,55,56]. Therefore, inadequate patient selection and/or surgical indication may negatively affect patients’ outcomes independent of their compensation status. 

Differences between clinical and work-related outcomes among WC and NWC patients may have multiple explanations and depend on both clinical and nonclinical factors. First, work accidents and/or occupational diseases usually have particularly serious adverse health consequences, and they are associated with high and severe degrees of temporary or permanent disability [57,58]. For example, WC patients are more likely to depend on opioids for pain relief [59] and present with worse symptoms, probably due to the increased injury severity in work environments [60]. The use of narcotics after occupational acute low back injury has been associated with an increased risk of chronic disability [2]. In a retrospective study by Anderson et al. [61], only 11% of WC subjects assuming chronic opioids (>1 year after surgery) sustainedly returned to work compared to individuals using opioids in the short post-operative term. Moreover, these patients showed an increased risk of psychiatric comorbidities, failed-back syndrome, and additional surgery, with substantially higher medical costs. In a recent study conducted by Kukreja and colleagues, 41.3% patients within a WC cohort underwent reoperation after lumbar discectomy and/or laminectomy following an on-the-job injury [62]. Thence, increased reoperation rate may additionally contribute to worsen surgical outcome and satisfaction in this population. 

Moreover, the relevance of the psychological status in patients undergoing lumbar spine surgery has been outlined by recent studies and may thus have a significant role in this specific subset of patients [63]. Indeed, WC subjects undergoing lumbar fusion and diagnosed with depression demonstrated higher rates of other psychiatric disorders, narcotic utilization and additional lumbar surgery compared to patients without depression. These individuals required significantly higher medical expenses due to their condition, with a very low RTW rate [64]. However, the aforementioned clinical factors are not sufficient on their own to explain why in WC subjects are observed worse results both in clinical and work-related terms.

Indeed, in this regard, the available literature data call into question also numerous nonclinical factors that mainly include demographic and socioeconomic variables such as male gender [65], lower degree of education [66], higher body mass index [67], smoking history [68], longer working hours [65], higher physical demands [69], civil litigation, legal representation [50,61,64], lower annual income and need for financial assistance [70,71]. Furthermore, longer compensation periods and higher compensation costs in WC patients may also depend on the fact that these subjects are more likely to conduct risky activities with higher chances of injury. A recent study by Khor et al. [72] proposed a prediction model for pain and functional outcomes following lumbar spine fusion surgery. Interestingly, they found that patients with worse improvements in pain and disability were more likely covered by WC and presenting with better preoperative ODI and NRS scores. In this regard, identifying presurgical risk factors and optimizing subject selection criteria for lumbar spine surgery in WC patients may help provide the most appropriate care for these individuals as well as to reduce the economic burden on national institutions providing WC. 

At the same time, disputed and complex claims also represent an impeding condition for a prompt RTW. Indeed, they induce a sort of conflict of interest in workers since it is not in the claimant’s interest to resume his working activity until the claim is resolved [70]. Several studies showed that a WC claim delays RTW [73,74]. In detail, data provided by our meta-analysis are in good agreement with previous published findings supporting the evidence that NWC returned fully to work at a faster rate than workers with recognized claims, especially after the request is denied [73]. However, studies on this topic commonly refer to NWC patients simply as individuals with no form of compensation, without specifying they did not possess the eligibility criteria or if, despite having made a claim, it was denied by the compensation authority. This is a substantial element to adequately understand the complex interaction between compensation status and health or work-related outcomes. Therefore, rather than comparing workers solely based on their compensation status, it would be useful to consider also claim processing time or any possible appeals made by workers in case of claim rejection. Indeed, some studies suggested that the observed negative association with the recognition of a compensation state could depend on an inefficient, long, and overly bureaucratic claim management [75]. Furthermore, claim processing times (and consequently RTW) might be also influenced by other factors related to the worker, workplace or the nature/severity of the work accident or occupational disease. For example, in the case of cause-based system compensations, it is not always easy or obvious to define a link between adverse effects suffered by workers and their working activities or exposure to certain occupational risk factors, especially when workers are elderly and have often important comorbidities [76,77]. 

On the other hand, it can be postulated that these patients, thanks to the financial support provided by WC and prolonged abstention from work, may be more likely to experience a full recovery without undertaking harmful activities.

This study has some limitations. Firstly, the overall level of evidence of the studies included is low due to the absence of RCTs comparing WC and NWC populations. Moreover, the NRCTs included were classified as “low quality” according to GRADE and single studies ranged from “low” to “high” risk of bias according to ROBINS-I. The small sample size of some included articles and the high heterogeneity among studies (I^2^ = 55%, 62%, 82% and 67% for pain, disability, RTW and satisfaction outcomes, respectively), downgraded the overall quality of our results and may have led to an overestimation of their effects. As observational studies constituted the main source of our analysis, selection bias and confounding due to diverse expectations in WC patients should be taken in consideration. In addition, the different definition of RTW and heterogeneous lengths of follow-up in the examined studies may generate further inconsistencies. Moreover, as regulations of WC in terms of expense coverage, compensation amount, claim duration profoundly differ among countries, it is difficult to generalize our results to all compensation systems [78]. This is particularly true when considering the extreme fragmentation of the American compensation systems, especially in terms of coverage, benefit adequacy, disability determination and complexity of claims [79]. Furthermore, having excluded studies in languages other than English and Italian could have limited our understanding of the relationship between WC and surgical outcomes in different nations. 

## 5. Conclusions

To our knowledge, this is the first systematic review and meta-analysis totally focused on the effect of WC on patients after lumbar spine surgery and the most updated report on the topic. Differently from previous studies, we have stratified post-operative outcomes in clinical (pain, disability, and satisfaction) and work-related (RTW) domains. This reflects the multifactorial nature of the phenomenon and may contribute to clarify which factors (and to what extent) are likely involved in reducing the clinical efficacy of surgery in such a specific population. Indeed, our findings are in good agreement with those already published in the literature, further confirming that the compensation status negatively affects both clinical and work-related outcomes. In this regard, the confounding bias induced by subjects receiving a compensation is a quite common drawback in lumbar spine surgery research investigating the effectiveness and the results of the therapeutic interventions adopted to deal with work-related diseases, conditions, and injuries. However, it is important to underline that it is not yet clear whether the negative effects on the different outcomes are a direct consequence of the compensation status itself or rather are more related to some specific aspects that are necessary to obtain the compensation status (i.e., time, claims, administrative and bureaucratic process). Therefore, it would be necessary to obtain a better understanding of the different aspects and intrinsic characteristics that govern the compensation recognition. In this regard, future studies on this topic should in our opinion focus not so much on the comparison between WC and NWC but rather on the analysis (within the WC group) of the different variables that can influence the timing and modalities with which the compensation status is recognized or not.

## Figures and Tables

**Table 1:** Main characteristics of the studies included in the meta-analysis.

Author	Year	Country	Type of Study	LOE	Sample Size WC	Sample Size NWC	Mean Age WC (y)	Mean Age NWC (y)	Mean Age (y)
Agazzi et al. [23]	1999	Switzerland	Retrospective	3	34	37	-	-	-
Albert et al. [24]	2000	USA	Retrospective	3	28	9	-	-	40.3 ± 10.3
Asch et al. [25]	2002	USA	Prospective	2	80	132	-	-	41 ± 11.3
Atlas et al. [26]	2000	USA	Prospective	2	56	120	38.7	41.2	-
Carreon et al. [27]	2010	USA	Case-control	3	58	58	47.9 ± 9.4	47.8 ± 9.4	-
Deutsch et al. [28]	2006	USA	Prospective	2	4	16	-	-	49
Greenough et al. [29]	1994	UK	Prospective	2	106	45	-	-	-
Greenough et al. [30]	1998	UK	Prospective	2	66	62	-	-	-
Gum et al. [16]	2013	USA	Case-control	3	38	38	42	42.2	-
Herron et al. [31]	1996	USA	Prospective	2	89	186	-	-	43 (15–83)
Klekamp et al. [32]	1998	USA	Retrospective	3	23	27	35.0 ± 7.1	39.5 ± 12.0	-
Lew et al. [33]	2001	USA	Retrospective	3	10	37	49.7 ± 9.8	50.7 ± 10.8	-
MacKay et al. [34]	1995	USA	Prospective	2	46	108	-	-	40 (20–79)
Madan et al. [18]	2003	UK	Prospective	2	12	27	-	-	43 (25–67)
Marks et al. [35]	2000	USA	Retrospective	3	51	51	-	-	-
Montgomery et al. [36]	2015	New Zealand	Prospective	2	120	49	53 (24–81)	61 (31–82)	-
Parker et al. [37]	1996	USA	Prospective	2	10	23	-	-	41 (22–56)
Pelton et al. [38] **^§^**	2012	USA	Prospective	2	11	22	-	-	51.7 ± 12.2
Pelton et al. [38] **^§^**	2012	USA	Prospective	2	13	20	-	-	49.9 ± 10.7
Penta et al. [39]	1997	Australia	Prospective	2	61	42	-	-	48 (28–73)
Phan et al. [40]	2017	Australia	Prospective	2	24	90	46.3 ± 10.4	60.2 ± 12.9	-
Rouben et al. [41]	2011	USA	Retrospective	3	14	155	-	-	44.5 ± 10.9
Sanderson et al. [42]	1999	Australia	Retrospective	3	12	12	-	-	33.1 ± 14.2
Schnee et al. [43]	1997	USA	Retrospective	3	20	32	-	-	53.4 (24–77)
Slosar et al. [44]	2000	USA	Retrospective	3	73	60	-	-	38.8 (21–58)
Taylor et al. [45]	2000	USA	Prospective	2	47	189	-	-	46
Vaccaro et al. [46]	1997	USA	Case series	4	13	11	37	39	38 (24–50)

^§^ The study from Pelton et al. is composed of two different cohorts as illustrated in the table. LOE = level of evidence; WC = workers’ compensation; NWC = non-worker’ compensation.

**Table 2:** Types of lumbar spine surgery, outcomes measured in WC and NWC and main findings obtained by the studies included in the meta-analysis.

Author	Last Follow-Up	Type of Surgery	Comparison	Outcomes Measures	Conclusions
Agazzi et al. [23]	2 y	PLIF	Yes (internal)	Prolo economic and functional scale WC: 16/34 (47%); NWC: 31/37 (84%) RTW WC 2/33; NWC 24/30	Socioeconomic factors and WC issues seem to be significant prognostic indicators of outcome.
Albert et al. [24]	2 y	Anteroposterior fusion	Yes (internal)	Functional status Success: WC 18/27; NWC 9/27 Failure: WC 10/12; NWC 2/12	WC increased the chance of functional failure, though this correlation was not statistically significant.
Asch et al. [25]	3 y	Microdiscectomy	Yes (internal)	Pain relief success rate WC 67.9%; NWC 82.8% (*p* < 0.05) ODI success rate (< 40%) WC 63.5%; NWC 86.5% (*p* < 0.001) Satisfaction after surgery WC 63.2%; NWC 83.5% (*p* < 0.001) RTW WC 42.1%; NWC 71.5% (*p* < 0.001)	Progressively poorer outcomes occur with increasing patient age up to the late-50 s and confirms the disparity in outcomes between cases in which WC is being sought and those in which it is not.
Atlas et al. [26]	4 y	Open discectomy, percutaneous discectomy	Yes	Relief from pain WC 20 (36%); NWC 81 (68%) RMDQ WC −9.3; NWC −12.5 Satisfaction WC 24 (43%); NWC 85 (71%) RTW WC 17/133 (13%); NWC 7/190 (4%)	Patients who have been receiving WC at baseline were more likely to be receiving disability benefits and were less likely to report relief from symptoms and improvement in quality of life at the time of the four-year follow-up than patients who had not been receiving WC at baseline. Nonetheless, most patients returned to work regardless of their initial disability status, and those who had been receiving WC at baseline were only slightly less likely to be working after four years.
Carreon et al. [27]	2 y	PLF	Yes	NRS back WC 1.7 ± 3.1; NWC 2.5 ± 2.7 (*p* = 0.073) ODI WC 4.9 ± 14.1; NWC 13.3 ± 17.1 (*p* = 0.009) SF-36 WC −1.3 ± 9.7; NWC 3.9 ± 8.9 (*p* = 0.007)	Patients on WC have significantly less improvement of clinical outcomes in both mean change in ODI and SF-36, as well as the number of patients achieving substantial clinical benefit.
Deutsch et al. [28]	1 y	Unilateral TLIF with PLF	Yes, but not clearly defined	ODI WC: 3/4 patients improved at 6 months RTW WC: 2/4	No differences between WC and NWC were reported concerning to disability and RTW.
Greenough et al. [29]	2 y	ALIF	Yes	Satisfaction after surgery (*p* < 0.05) 8–10: WC (35; 37%); NWC (28; 67%) 5–7: WC (37; 40%); NWC (8; 19%) 2–4: WC (18; 19%); NWC (3; 7%) 0–1: WC (4; 4%); NWC (3; 7%) LBOS (*p* < 0.01) Excellent: WC (8; 10%); NWC (13; 43%) Good: WC (19; 22%); NWC (10; 25%) Fair: WC (34; 40%); NWC (10; 25%) Poor: WC (24; 28%); NWC (7; 17%)	The rate of fusion was influenced by the presence of a WC claim. WC status and psychological disturbance at presentation were significant prognostic factors. Psychological disturbance at review had a profound effect on the outcome and patient satisfaction ratings.
Greenough et al. [30]	2 y	PLF	Yes	LBOS WC 25 (7–72, *n* = 57); NWC 35 (7–75, *n* = 63) *p* < 0.001 Satisfaction after surgery WC 2 (0–3, *n* = 56); NWC 2 (0–3, *n* = 59) *p* < 0.02 VAS WC 6 (1–10, *n* = 57); NWC 5 (0–10, *n* = 62) *p* < 0.02	Results of instrumented PLF are poor and indications for the procedure need careful consideration. The results are significantly influenced by WC but not by technical success.
Gum et al. [16]	2 y	TLIF or PLF	Yes	VAS WC 0.94; NWC 2.51 (*p* = 0.011) ODI mean change WC 5.54; NWC 15.17 (*p* = 0.009) SF-36 mean change WC 1.69; NWC 4.09 (*p* = 0.235)	Patients receiving WC have the perception of poor clinical outcomes after lumbar fusion.
Herron et al. [31]	4 y	Laminectomy and discectomy	Yes (internal)	Surgical outcome (*p* = 0.00) Good: WC 52 (58%); NWC 174 (94%) Fair: WC 16 (18%); NWC 10 (5%) Poor: WC 21 (24%); NWC 2 (1%)	Patients with WC or litigation issues were significantly more likely to have poor outcomes.
Klekamp et al. [32]	11 m	Discectomy	Yes	WC: 29% of patients achieved good results NWC: 81% of patients achieved good results	WC group achieved worse results compared to NWC group.
Lew et al. [33]	18 m (4–51 m)	Discectomy	Yes	Satisfaction after surgery (%) Excellent or good WC: 7 (70); NWC: 33 (89) *p* = 0.12 Excellent WC: 5 (50); NWC: 22 (60) *p* = 0.24 Good WC: 2 (20); NWC: 11 (30) *p* = 0.27 Fair WC: 0 (0); NWC: 2 (5.4) *p* = 0.62 Poor WC: 3 (30); NWC: 2 (5.4) *p* = 0.05 RTW WC: 90%; NWC: 93% *p* = 0.45	WC recipients experienced significantly worse outcomes than the other patients in this study. Nevertheless, a high RTW rate was maintained (90%) in both groups.
MacKay et al. [34]	1 y	Hemilaminotomy, discectomy	Yes	Prolo scale Satisfactory: WC: 63%; NWC: 92% (*p* < 0.0001) Unsatisfactory: WC: 37%; NWC: 8%	WC group had a lower success rate compared to NWC group.
Madan et al. [18]	2.4 y (2–3.1 y)	PLF and PLIF	Yes (internal)	ODI ALIF group (*p* = 0.0056) Satisfied WC: 6 (50); NWC: 25 (92.6) Unsatisfied WC: 6 (50); NWC: 2 (7.4) PLIF (*p* = 0.0064) Satisfied WC: 4 (45); NWC: 24 (92.3) Unsatisfied WC: 5 (55.5); NWC 2 (7.7)	There were no differences between WC and NWC groups concerning to disability.
Marks et al. [35]	30.7 ± 17.9 m	Percutaneous discectomy	Yes, but not clearly defined	Pain, Job function, Physical restrictions, medications WC and NWC no differences (data not available), *p* > 0.05	WC status does not influence the outcomes.
Montgomery et al. [36]	8 y (4–14 y)	Lumbar spinal fusion	Yes	RMDQ 1-year postoperative WC: 8.0, 6.8–9.2/NWC: 4.6, 2.8–6.5 (*p* < 0.05) At long-term follow-up WC: 5.9, 4.7–7.1/NWC: 3.8, 1.9–5.8 (*p* > 0.05) LBOS 1 year post-operative WC: 43.9, 39.9–48.0/NWC: 54.1, 48.4–59.9 (*p* < 0.05) Long term follow-up WC: 47.0, 43.5–50.4/NWC: 55.4, 49.3–61.6 (*p* > 0.05) SF12 Long term follow up WC 41.6 ± 11.5/NWC 44.0 ± 13.0 (*p* > 0.05)	ACC patients achieved equivalent improvements compared to non-ACC patients and NWC patients as per in the published literature. They also achieve function that is considerably better than that achieved in WC patients in adversarial compensation jurisdictions.
Parker et al. [37]	47 m (27–84 m)	PLF	Yes	Clinical outcome pain, medications, and resume of previous activities WC: 1/10 good, 9/10 poor results NWC: 9/23 good or excellent, 3/23 fair, 11/23 poor results	Patients in WC group showed worse clinical outcomes compared to NWC group.
Pelton et al. [38]	6 m	MIS-TLIF and open TLIF	Yes	VAS (MIS-TLIF cohort) Differences between WC and NWC (*p* = 0.712) VAS (open TLIF cohort) Differences between WC and NWC (*p* = 0.241)	Immediate outcomes and hospitalizations between NWC and WC populations did not differ regardless of surgical technique (MIS/open). Differences occurred in improved outcomes with an MIS-TLIF versus an open TLIF even in a WC environment.
Penta et al. [39]	10 y	ALIF	Yes	LBOS WC: 38 (4–74); NWC: 45 (11–75) (*p* = 0.06) VAS WC: 4 (0–9), NWC: 4 (0–10) (*p* = 0.29)	WC had a negative effect on outcomes only in the first period (two years). After 10 years of follow up this effect disappeared.
Phan et al. [40]	2 y	ALIF	Yes	SF-12 WC: 11.3; NWC: 9.1 (*p* = 0.691) ODI WC: 26.3; NWC: 33.4 (*p* = 0.232)	No significant differences found between WC and NWC patients in terms of fusion rates, complications, clinical outcomes.
Rouben et al. [41]	50 m	MIS-TLIF	Yes (internal)	RTW 57% of WC patients (mean time: 17 weeks) ODI Mean change of 34% (*p* < 0.001) Post-operative VAS Significant improvement WC patients (*p* < 0.001)	WC patients responded well to surgical treatment.
Sanderson et al. [42]	3.1 y	Short segment fixation	Yes	LBOS WC: 45.1; NWC: 67.4 (*p* < 0.05)	The presence of a WC claim positively influenced the outcomes after surgery.
Schnee et al. [43]	18.6 m (6–36.7 m)	PLF	Not clearly defined	RTW WC: not defined; NWC: 84% of cases; Prolo scale Significant adverse effects of WC (*p* = 0.0001). Good pain results were seen in 81% of NWC	WC claims and smoking had very significant adverse impacts on both employment and pain results despite high fusion rates, particularly in patients under the age of 55.
Slosar et al. [44]	37.2 m	ALIF + PLF	Yes, but not clearly defined	Satisfaction after surgery (*p* > 0.05) 1 (best): WC 7 (9.6%); NWC 7 (11.7%) 2: WC 36 (49.3); NWC 32 (53.3%) 3: WC 14 (19.1%); NWC 12 (20%) 4: WC 16 (22%); NWC 9 (15%)	There was not a statistically significant difference in terms of satisfaction following surgery between WC and NWC patients.
Taylor et al. [45]	18 m	Discectomy, laminectomy, or fusion	Not clearly defined	Much better functioning WC: 52%; NWC: 68% *p* < 0.05 Very positive about the treatment WC: 57%; NWC: 71% *p* < 0.05	The study results indicate that WC payments and litigation are two important predictors of poor outcomes after low back surgery in community practice.
Vaccaro et al. [46]	37 m (18–64 m)	Uninstrumented PLF	No	Satisfaction after surgery Fair/poor results: WC: 13; NWC: 2 RTW None of the WC patients returned to work	WC is strongly associated with poor results of operative management of LBP in adult patients with low-grade spondylolisthesis.

ACC = Accident Compensation Corporation; ALIF = anterior lumbar interbody fusion; LBOS = low back outcome score; MIS = minimally invasive surgery; NRS = numeric rating scale; NWC = non-worker’ compensation; ODI = Oswestry Disability Index; PLF = posterolateral fusion; PLIF = posterolateral interbody fusion; RMDQ = Roland and Morris Disability Questionnaire; RTW = return to work; SF-12 = 12-item Short Form Health Survey; SF-36 = 36-item Short Form Health Survey; TLIF = transforaminal lumbar interbody fusion; VAS = visual analogue scale; WC = workers’ compensation.

## Data Availability

The datasets used and/or analyzed during the current study are available from the corresponding author on reasonable request.

## Supplementary Materials

The following are available online at https://www.mdpi.com/article/10.3390/ijerph18116165/s1, Figure S1: Methodological quality assessments of each study assessed using ROBINS, Table S1: GRADE profile of evidence, Table S2: GRADE Summary of findings.
