# Supplementary material for: Does Workers’ Compensation Status Affect Outcomes after Lumbar Spine Surgery? A Systematic Review and Meta-Analysis
Source: Int J Environ Res Public Health. 2021 Jun 7;18(11):6165. doi: 10.3390/ijerph18116165 (PMC8201180; doi:10.3390/ijerph18116165)
Supplement: Supplementary file 1 [file ijerph-18-06165-s001.zip › Supplementary Figure S1.pdf]

| | D1 | D2 | D3 | D4 | D5 | D6 | D7 | Overall* |
|---|---|---|---|---|---|---|---|---|
| Agazzi 1999 | ? | ? | + | + | + | ? | − | − |
| Albert 2000 | ? | + | + | ? | + | ? | + | ? |
| Asch 2002 | + | + | + | + | + | + | + | + |
| Atlas 2000 | ? | ? | ? | + | + | ? | ? | ? |
| Carreon 2010 | ? | + | + | ? | ? | + | + | ? |
| Deutsch 2006 | + | + | + | ? | + | ? | ? | ? |
| Greenough 1994 | + | + | + | + | + | + | + | + |
| Greenough 1998 | + | + | + | + | + | + | + | + |
| Gum 2013 | + | + | + | ? | + | ? | ? | ? |
| Herron 1996 | + | + | + | ? | + | ? | ? | ? |
| Klekamp 1998 | + | + | ? | ? | + | + | + | ? |
| Lew 2001 | + | + | ? | ? | + | + | ? | ? |
| Mackay 1995 | + | + | + | + | + | + | + | + |
| Madan 2003 | + | + | + | + | + | + | + | + |
| Marks 2000 | + | + | + | + | + | + | + | + |
| Montgomery 2015 | − | + | ? | + | + | + | − | − |
| Parker 1996 | + | + | + | + | + | + | − | − |
| Pelton 2012 | + | + | + | + | + | ? | + | ? |
| Penta 1997 | ? | + | ? | + | ? | + | − | − |
| Phan 2017 | + | + | + | + | + | + | + | + |
| Rouben 2011 | + | + | + | + | ? | + | ? | ? |
| Sanderson 1999 | + | + | + | + | + | + | ? | ? |
| Schnee 1997 | ? | + | + | + | + | + | ? | ? |
| Slosar 2000 | ? | + | + | + | + | ? | − | − |
| Taylor 2000 | ? | + | + | + | + | ? | ? | ? |
| Vaccaro 1997 | ? | + | + | + | + | ? | ? | ? |
