# Supplementary material for: Does Workers’ Compensation Status Affect Outcomes after Lumbar Spine Surgery? A Systematic Review and Meta-Analysis
Source: Int J Environ Res Public Health. 2021 Jun 7;18(11):6165. doi: 10.3390/ijerph18116165 (PMC8201180; doi:10.3390/ijerph18116165)
Supplement: Supplementary file 1 [file ijerph-18-06165-s001.zip › Supplementary Table S1.pdf]

International Journal of
Environmental Research and Public Health

MDPI

Supplementary Table S1. GRADE Profile of evidence

Certainty assessment							N° of patients		Effect		Certainty
N° of studies	Study design	Risk of bias	Inconsistency	Indirectness	Imprecision	Other considerations	WC	NWC	Relative (95% CI)	Absolute (95% CI)	
**Pain**[16,25-27,32,41]											
6	Observational studies	Serious	Serious[a]	Not serious	Not serious	None	137/270 (50.7%)	109/531 (20.5%)	**RR 1.79** (1.32 to 2.42)	16 more per 100 (from 7 more to 29 more)	⊕⊕◯◯ LOW
**Disability**[16,18,24,25,27,28,36,40,41,45]											
10	Observational studies	Serious	Serious[b]	Not serious	Not serious	None	438/666 (65.8%)	347/1028 (33.8%)	**RR 1.38** (1.17 to 1.63)	13 more per 100 (from 6 more to 21 more)	⊕⊕◯◯ LOW
**Return to work**[25,26,28,29,33,41,43,44,46]											
9	Observational studies	Serious	Serious[b]	Not serious	Not serious	None	182/355 (51.3%)	216/915 (23.6%)	**RR 1.68** (1.41 to 1.99)	161 more per 1.000 (from 97 more to 234 more)	⊕⊕◯◯ LOW
**Satisfaction**[23,25,26,29-31,33-35,37,39,43-46]											
15	Observational studies	Serious	Serious[c]	Not serious	Not serious	None	303/727 (41.7%)	221/1114 (19.8%)	**RR 2.10** (1.82 to 2.44)	218 more per 1.000 (from 163 more to 286 more)	⊕⊕◯◯ LOW

RR: Risk ratio; Explanations: a. I^2=55%, Chi^2 p value = 0.07, b. I^2=8
