# Supplementary material for: Does Workers’ Compensation Status Affect Outcomes after Lumbar Spine Surgery? A Systematic Review and Meta-Analysis
Source: Int J Environ Res Public Health. 2021 Jun 7;18(11):6165. doi: 10.3390/ijerph18116165 (PMC8201180; doi:10.3390/ijerph18116165)
Supplement: Supplementary file 1 [file ijerph-18-06165-s001.zip › Supplementary Table S2.pdf]

International Journal of Environmental Research and Public Health

MDPI

Supplementary Table S2. GRADE Summary of findings

Outcomes	Anticipated absolute effects* (95% CI)		Relative effect (95% CI)	N° of participants (studies)	Certainty of the evidence (GRADE)
	Risk with NWC	Risk with WC			
Pain	20 per 100	37 per 100 (27 to 50)	**RR 1.79** (1.32 to 2.42)	801 (6 observational studies)	⊕⊕◯◯ LOW [a]
Disability	34 per 100	47 per 100 (39 to 55)	**RR 1.38** (1.17 to 1.63)	1694 (10 observational studies)	⊕⊕◯◯ LOW [b]
Return to work	236 per 1.000	397 per 1.000 (333 to 470)	**RR 1.68** (1.41 to 1.99)	1270 (9 observational studies)	⊕⊕◯◯ LOW [c]
Satisfaction	198 per 1.000	417 per 1.000 (361 to 484)	**RR 2.10** (1.82 to 2.44)	1841 (15 observational studies)	⊕⊕◯◯ LOW [d]

*The risk in the intervention group (and its 95% confidence interval) is based on the assumed risk in the comparison group and the relative effect of the intervention (and its 95% CI).
CI: Confidence interval; RR: Risk ratio

Explanations: a. $I^2 = 55\%$, Chi^2 *p* value = 0.07, b. $I^2 = 82\%$, c. $I^2 = 67\%$
